# FPGA Implementation of Efficient CFAR Algorithm for Radar Systems

**DOI:** 10.3390/s23020954

**Published:** 2023-01-13

**Authors:** Yunseong Sim, Jinmoo Heo, Yongchul Jung, Seongjoo Lee, Yunho Jung

**Affiliations:** 1 School of Electronics and Information Engineering, Korea Aerospace University, Goyang-si 10540, Republic of Korea; 2 Department of Smart Air Mobility, Korea Aerospace University, Goyang-si 10540, Republic of Korea; 3 Korea Electronics Technology Institute (KETI), Bundang, Seongnam 13509, Republic of Korea; 4 Department of Information and Communication Engineering, Sejong University, Seoul 05006, Republic of Korea; 5 Department of Convergence Engineering of Intelligent Drone, Sejong University, Seoul 05006, Republic of Korea

**Keywords:** radar signal processing, constant false alarm rate, target detection, field programmable gate array, automotive radar, drone detection radar

## Abstract

The constant false-alarm rate (CFAR) algorithm is essential for detecting targets during radar signal processing. It has been improved to accurately detect targets, especially in nonhomogeneous environments, such as multitarget or clutter edge environments. For example, there are sort-based and variable index-based algorithms. However, these algorithms require large amounts of computation, making them difficult to apply in radar applications that require real-time target detection. We propose a new CFAR algorithm that determines the environment of a received signal through a new decision criterion and applies the optimal CFAR algorithms such as the modified variable index (MVI) and automatic censored cell averaging-based ordered data variability (ACCA-ODV). The Monte Carlo simulation results of the proposed CFAR algorithm showed a high detection probability of 93.8% in homogeneous and nonhomogeneous environments based on an SNR of 25 dB. In addition, this paper presents the hardware design, field-programmable gate array (FPGA)-based implementation, and verification results for the practical application of the proposed algorithm. We reduced the hardware complexity by time-sharing sum and square operations and by replacing division operations with multiplication operations when calculating decision parameters. We also developed a low-complexity and high-speed sorter architecture that performs sorting for the partial data in leading and lagging windows. As a result, the implementation used 8260 LUTs and 3823 registers and took 0.6 μs to operate. Compared with the previously proposed FPGA implementation results, it is confirmed that the complexity and operation speed of the proposed CFAR processor are very suitable for real-time implementation.

## 1. Introduction

**Figure 1:**
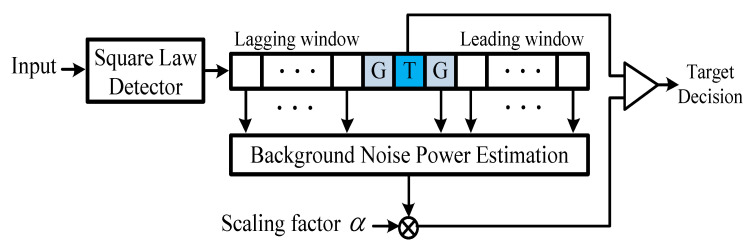
Block diagram of CFAR detector.

The CFAR algorithm, which detects targets using variable thresholds, is essential in radar signal processing. In general, if the amplitude of the received signal is higher than the threshold, it is determined as a target. However, it is designated as noise when the amplitude of the received signal is lower than the threshold. If the threshold is constant, the target is not recognized, and the false alarm rate rapidly increases. The CFAR algorithm generates appropriate thresholds depending on the amplitude of the received signal to increase the probability of detection and reduce the false alarm rate. The architecture of the CFAR algorithm is illustrated in Figure 1 [1,2]. The window of the CFAR algorithm comprises a test cell, a guard cell, and a reference window. The reference window consists of a leading window and a lagging window that surround the guard cell. The power of the background noise to detect the target is estimated by the reference window. After estimating the background noise power, the final threshold is calculated by multiplying the background noise by a scaling factor for the desired false alarm rate. The threshold determines whether the test cell is being targeted.

A classic CFAR algorithm is the cell averaging (CA) CFAR algorithm. The CA CFAR algorithm performs best in a homogeneous environment where the noise follows an exponential distribution and the noise samples are independently and identically distributed (IID) [2]. However, there is a problem in that the performance rapidly degrades in a nonhomogeneous environment. In most cases, the received signal is nonhomogeneous, and representative nonhomogeneous environments are the clutter edge environment and the multitarget environment [3]. The clutter edge environment is caused by changes in weather, jamming, and sudden changes in elevation/reflection [4,5]. The multitarget environment involves cases in which interfering targets exist in the reference window but not in the test cell [6]. For the CA CFAR algorithm, the threshold is calculated using the average of both the leading and lagging windows; therefore, if there is an interfering target, the threshold increases, and the detection probability decreases [7,8,9].

Greatest of CFAR (GO CFAR), smallest of CFAR (SO CFAR), and ordered statistic CFAR (OS CFAR) have been proposed to overcome the nonhomogeneous environment problem [3,10,11]. However, they only perform well in specific nonhomogeneous environments [5]. For GO CFAR, the performance improves in a clutter edge environment, but there is degradation in multitarget environments. SO CFAR and OS CFAR have improved performance in multitarget environments, but their performance degrades in clutter edge environments. Thus, the variability index (VI) CFAR was proposed to apply the optimal CFAR algorithm by analyzing the environment to improve the performance in all nonhomogeneous environments [5]. After analyzing the environment, VI CFAR uses GO CFAR in a clutter edge environment and applies SO CFAR in a multitarget environment. However, if there is an interfering target in both the leading and lagging windows, the performance of SO CFAR degrades.

Algorithms to improve this performance in a multitarget environment have been proposed. Improved version of the VI-CFAR (IVI CFAR), switching variability index CFAR (SVI CFAR), and modified VI CFAR (MVI CFAR) algorithms have been proposed to improve the performance of multitarget environments in VI CFAR [12,13,14]. IVI CFAR and SVI CFAR improve multitarget performance by applying OS CFAR and switching (S) CFAR. MVI CFAR applies trimmed mean (TM) CFAR to multitarget environments, and the subwindow is applied to the clutter edge environment. In addition, the sort-based algorithm sparsity adaptive correlation maximization (SACM) CFAR, first-order difference FOD) CFAR, between-class variance (BCV) CFAR, and automatic censored cell averaging-based ordered data variability (ACCA-ODV) CFAR have been proposed [15,16,17,18].

Although there are studies on the CFAR algorithm to improve detection performance in various environments, they have very high computation requirements in most cases. Therefore, CFAR algorithms used for real-time detection, such as radar signal processing, need hardware design for practical applications. SACM CFAR, FOD CFAR, and BCV CFAR perform alignment and require large amounts of computation to maintain a high detection probability [19]. Therefore, we developed a novel CFAR algorithm that achieves excellent performance in both homogeneous and nonhomogeneous environments by utilizing MVI CFAR with improved performance in clutter edge environment and ACCA-ODV CFAR with good detection probabilities in multitarget environment. We also designed an optimal hardware structure to perform real-time tasks. To reduce hardware complexity, the division operation was replaced by multiplication, and the time-sharing sum and square operations were used. We also applied a low-complexity and high-speed sorter architecture to sort the partial data in leading and lagging windows.

The main contribution of this study can be summarized as follows: (1) We propose an efficient CFAR algorithm that can support superior performance for homogeneous and nonhomogeneous environments owing to a new decision criterion with MVI and ACCA-ODV CFAR algorithms; (2) the low-complexity and high-speed hardware architecture of the proposed CFAR processor and the real-time implementation results based on the FPGA device are presented. The remainder of this paper is organized as follows: Section 2 describes the ACCA-ODV CFAR and MVI CFAR algorithms. Section 3 describes the proposed CFAR algorithm in detail, and a performance analysis is presented in Section 4. Section 5 discusses the optimum CFAR architecture, and Section 6 concludes the paper.

## 2. Previously Proposed CFAR Algorithms

### 2.1. ACCA-ODV CFAR

The ACCA-ODV CFAR algorithm is suitable for multitarget environments because it adaptively censors the high amplitude of the reference window according to the received signal and then calculates the threshold. However, its performance degrades in clutter edge environments because it sorts the reference window and adaptively censors cells that exhibit high amplitudes [20,21].

First, if the total reference window size is *N*, and X(i) is the value of the *i*th reference cell, the reference window is sorted in ascending order, as shown in Equation (1).

(1) X(1)≤X(2)≤⋯≤X(p)≤⋯≤X(N)

After sorting, the mean and variance are calculated as in Equations (2) and (3), respectively.

(2) $$\sigma_{p}=\sum_{i=1}^{p}X\left(i\right)$$

(3) $$\mu_{p}=\sum_{i=1}^{p}X{\left(i\right)}^{2}$$

where *p* denotes the number of small values required to determine homogeneity; $V_{k}$, which represents the degree of homogeneity, is calculated using X(N−k). The formula $V_{k}$ is as follows:

(4) $$V_{k}=\frac{\mu_{p}+{\left(X\left(N-k\right)\right)}^{2}}{{\left(\sigma_{p}+X\left(N-k\right)\right)}^{2}}$$

Finally, the homogeneity of {X(1),X(2),…,X(p),X(N−k)} is determined by comparing the calculated $V_{k}$ and $S_{k}$, which is the threshold value of $V_{k}$. If $V_{k}$ is less than $S_{k}$, it is nonvariable; if $V_{k}$ is greater than $S_{k}$, it is variable. To determine $S_{k}$, the probability of determining a homogeneous environment as a nonhomogeneous environment ($\alpha_{k}$) is experimentally obtained by comparing $S_{k}$ and $V_{k}$.

(5) $$\alpha_{k}=Prob\left(V_{k}>S_{k}|E_{k=X(N-k)}\;is\;homogeneous\right)$$

Thereafter, $S_{k}$ is obtained; it corresponds to an allowable parameter $P_{fc}$ for determining a homogeneous environment as a nonhomogeneous environment. The $S_{k}$ values were defined by simulations according to *N*, *p*, and $P_{fc}$[18]. If the dataset is deemed nonhomogeneous, *k* is increased by 1 and the above procedure is repeated until it is considered homogeneous. However, if it is determined to be homogeneous, the sum of {X(1),X(2),…,X(p),X(N−k)} is multiplied by the scaling factor $T_{k}$ to calculate a threshold and determine whether the target exists in the test cell.

### 2.2. VI CFAR and MVI CFAR

The VI CFAR algorithm computes the variability index (VI) and mean ratio (MR) to determine the environment of the received signal and calculates the threshold accordingly. VI, which comprises variance $\hat{\sigma^{2}}$ and mean $\hat{\mu }$, determines the homogeneity of the reference window. The VI CFAR computes the VI for each leading and lagging window to determine whether the window is nonhomogeneous. The formula for VI is shown in Equation (6), where $\bar{X}$ is the average of the window, and *N* is the size of the window.

(6) $$VI=1+\frac{\hat{\sigma^{2}}}{\hat{\mu^{2}}}=1+\frac{1}{\frac{N}{2}-1}\sum_{i=1}^{\frac{N}{2}}{\left(X\left(i\right)-\bar{X}\right)}^{2}/{\left(\bar{X}\right)}^{2}$$

To reduce the computational complexity of the VI formula in (6), it can be expressed as Equation (7).

(7) $$VI^{*}=1+\frac{1}{\frac{N}{2}}\sum_{i=1}^{\frac{N}{2}}{\left(X\left(i\right)-\bar{X}\right)}^{2}/{\left(\bar{X}\right)}^{2}=\frac{N}{2}\sum_{i=1}^{\frac{N}{2}}\left(x{\left(i\right)}^{2}\right)/(\sum_{i=1}^{\frac{N}{2}}X\left(i\right){)}^{2}$$

The calculated VI is compared with the threshold $S_{VI}$ to determine whether the window is homogeneous. It is variable when VI is less than $S_{VI}$ and nonvariable when VI is greater than $S_{VI}$.

MR is the mean ratio of the leading and lagging windows. As the number of cells in both windows is the same, it can be written as the sum ratio of each window.

(8) $$MR=\sum_{i=1}^{\frac{N}{2}}X\left(i\right)/\sum_{i=\frac{N}{2}+1}^{N}X\left(i\right)$$

The calculated MR is compared with $S_{MR}$ to determine whether the average of the leading and lagging windows is different. If MR is the same as Equation (9), it is considered a situation with the same average, and if it is the same as Equation (10), it is considered to have a different average.

(9) $$S_{MR}^{-1}\leq MR\leq S_{MR}$$

(10) $$MR<S_{MR}^{-1}\;or\;S_{MR}<MR$$

The probability of discriminating a homogeneous environment from a nonhomogeneous environment ($\alpha_{0}$) was experimentally obtained to determine $S_{VI}$.

(11) $$\alpha_{0}=Prob\left(Vi>S_{VI}|homogeneous\right)$$

Similarly, $S_{MR}$ experimentally obtained the probability of determining that the mean of both side reference windows ($\beta_{0}$) is different, even though the experimental environment was homogeneous.

(12) $$\beta_{0}=1-Prob\left(1/S_{MR}\leq MR\leq S_{MR}|homogeneous\right)$$

After VI and MR are computed to determine the environment of the received signal, the CA CFAR, GO CFAR, and SO CFAR algorithms are executed according to the environment determined.

The VI CFAR algorithm reduces the false alarm rate by executing GO CFAR when a clutter edge exists in the reference window. However, the detection probability is reduced because the GO CFAR algorithm computes the threshold by selecting the larger sum of the leading or lagging window. Furthermore, the VI CFAR algorithm shows a high detection probability using SO CFAR when the interfering target is in the leading or lagging window. However, when the interfering targets are in both windows, the detection probability degrades.

To solve these problems, MVI CFAR was proposed. The MVI CFAR algorithm adds a subwindows smaller than half the reference window size and uses it when the received signal is determined as the clutter edge. It estimates the location of the clutter edge in the reference window by calculating the MR of the sub-window. If the MRs in the subwindows are the same, the smaller VI window of the leading and locking windows are used to find the clutter edge. In addition, if all subwindows are determined as nonhomogeneous, the trimmed mean (TM) CFAR algorithm is executed instead of SO CFAR. However, in the case of a multitarget environment, a TM CFAR algorithm that censors a predetermined number of cells is applied, so if it does not fit the actual operating environment, a rapid performance degradation occurs [14,16].

## 3. Proposed CFAR Algorithm

The proposed CFAR algorithm determines the environment of the received signal using new decision criteria. The VI or MVI CFAR algorithm computes the VI of the leading and lagging windows and determines whether the reference window is homogeneous. However, they do not correctly determine the received signal environment. The proposed algorithm divides the window number of *M*, as shown in Algorithm 1, calculates the VI for each *M* window, and then determines the homogeneity.

**Figure 2:**
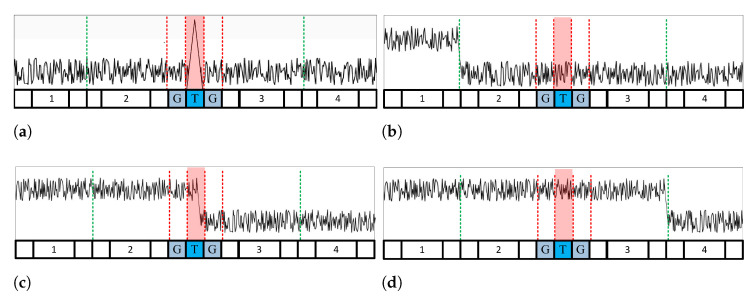
A case where no window is judged as variable: (**a**) homogeneous environment. (**b**) Clutter edge is located in window 1. (**c**) Clutter edge is adjacent to the test cell. (**d**) Cutter edge is located in window 4.

For example, if the entire reference cell is divided into four windows, as shown in Figure 2, the VIs in each of the four windows are calculated, and then each cell is determined, as shown in Equation (13).

(13) $$VI_{L}=\frac{N}{M}\sum_{i=\frac{N}{M}\left(L-1\right)+1}^{\frac{LN}{M}}\left(X{\left(i\right)}^{2}\right)/(\sum_{i=\frac{N}{M}\left(L-1\right)+1}^{\frac{LN}{M}}X\left(i\right){)}^{2}$$

If all the divided windows are determined to be homogeneous, as shown in Figure 2, the environment of the received signal is a homogeneous or cluttered edge. Then, the MR for the leading and lagging windows is calculated to determine whether a clutter edge exists in the reference window. If the two windows have the same mean, the environment is determined to be homogeneous. In contrast, if both windows have different means, they are determined by the clutter edge environment, as shown in Figure 2b–d. The threshold is generated differently depending on the clutter edge location.

When determined as a clutter edge environment, the $MR_{partition}$ is first calculated to estimate the position of the clutter edge. $MR_{partition}$ is calculated using the two windows closest to the test cell, as shown in Equation (14).

(14) $$MR_{partition}=\sum_{i=\frac{N}{2}-\frac{N}{M}+1}^{\frac{N}{2}}X\left(i\right)/\sum_{i=\frac{N}{2}+1}^{\frac{N}{2}+\frac{N}{M}}X\left(i\right)$$

For example, in Figure 2, $MR_{partition}$ calculates Windows 2 and 3 to determine the clutter edge. Figure 2c shows the case where $MR_{partition}$ determines that the mean of the two split windows is different. The GO CFAR algorithm is used because a clutter edge exists in the window adjacent to the test cell. However, if the two divided windows have the same mean, as shown in Figure 2b,d, there is no clutter edge in the window adjacent to the test cell. Therefore, CA CFAR is used by selecting a smaller VI window between the leading and lagging windows. In Figure 2b, CA CFAR uses a leading window because the VI of the leading window is smaller; in Figure 2d, CA CFAR uses a leading window because the VI of the lagging window is smaller.
**Algorithm 1** Pseudocode of the Proposed CFAR.**function**Proposed CFAR   **if** every window is nonvariable **then**   **if** result of MR is different mean **then**      **if** result of $MR_{partition}$ is different mean **then**      GO CFAR;      **else**      **if** leading window has smaller VI **then**         CA CFAR with leading window;      **else**         CA CFAR with lagging window;      **end if**      **end if**   **else**      CA CFAR;   **end if**   **else if** a window is variable **then**   **if** result of VL is lagging window **then**      CA CFAR with lagging window;   **else**      CA CFAR with leading window;   **end if**   **else if** if over 2 windows are variable **then**   ACCA-ODV CFAR;   **end if****end function**

**Figure 3:**
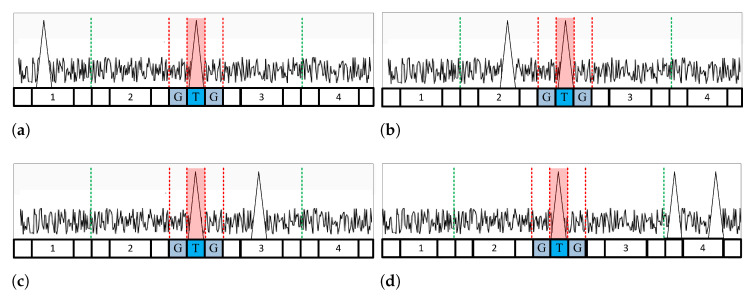
A case where a window is determined as a multitarget environment: interfering target is located in (**a**) window 1; (**b**) window 2. (**c**) window 3. (**d**) window 4.

**Figure 4:**
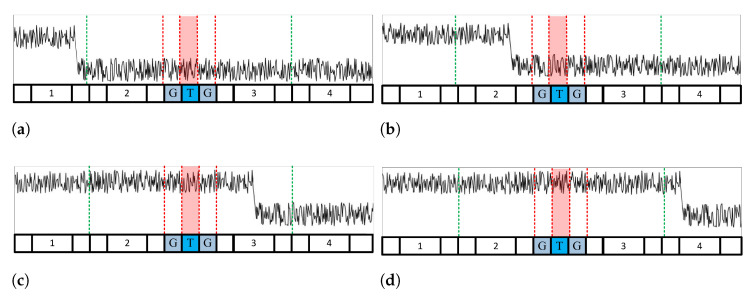
A case where a window is determined as a clutter edge environment: clutter edge is located in (**a**) window 1; (**b**) window 2; (**c**) window 3; (**d**) window 4.

When one window is deemed nonhomogeneous, a threshold value is generated without a nonhomogeneous window. For example, in multitarget environments, as shown in Figure 3, a threshold is generated using a window where interfering targets do not exist. When some clutter edges are entered, as shown in Figure 4, a threshold is generated using a part without the clutter edge. The variable window location ($V_{L}$) is estimated using the VI to classify the location of the nonhomogeneous window. As shown in Equation (15), if $V_{L}$ is less than half the total number of divided windows, the CA CFAR algorithm using the leading window is performed. As shown in Equation (16), the CA CFAR algorithm using a lagging window is performed if it is larger than half.

(15) $$1\leq V_{L}\leq \frac{M}{2}$$

(16) $$\frac{M}{2}+1\leq V_{L}\leq M$$

**Figure 5:**
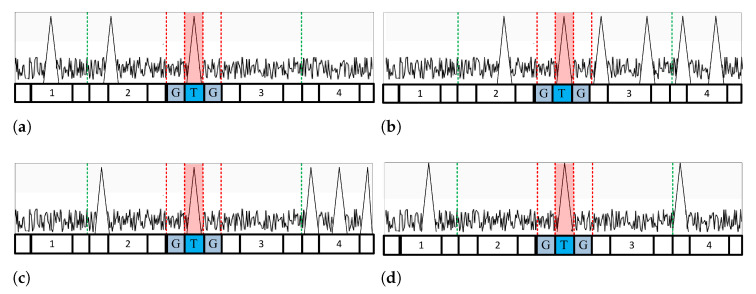
A case where two or more windows are determiend to be variable: interfering targets are in (**a**) windows 1 and 2; (**b**) windows 2–4; (**c**) windows 2 and 4; (**d**) windowd 1 and 4.

Figure 3 shows the multitarget environment: Figure 3a,b depict CA CFAR using the leading window, and Figure 3c,d depict CA CFAR using a lagging window. Figure 4 shows the clutter edge environments where a suitable CA CFAR algorithm is applied according to the clutter edge position. Figure 5 shows multitarget environments with two or more windows determined as being nonhomogeneous. Therefore, an ACCA-ODV CFAR algorithm that exhibits excellent performance in a multitarget environment is applied.

## 4. Performance Analysis

**Figure 6:**
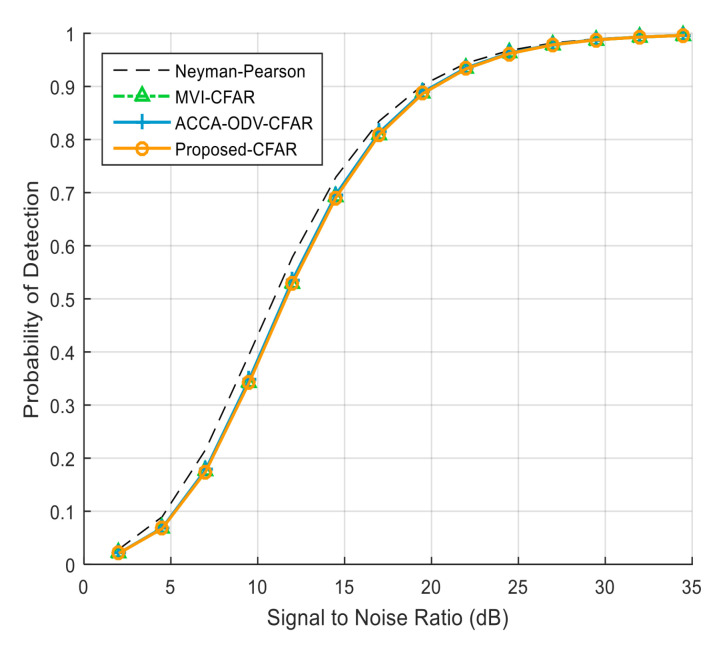
Detection probability in homogeneous environment (*N* = 36; $P_{fa}$ = $10^{-4}$).

The experiments were performed with Monte Carlo simulations using the parameters listed in Table 1. The total reference window size was 36, and 1 guard cell was located next to each side of the test cell. The false alarm probability ($P_{fa}$) was $10^{-4}$, and probability of false censoring ($P_{fc}$) was $10^{-2}$. The carrier-to-noise ratio (CNR) was 10 dB, the noise signal had an exponential distribution, and the noise power was assumed to be 1 dB. As illustrated in Figure 6, ACCA-ODV CFAR, MVI CFAR, and the proposed CFAR algorithm showed high detection probabilities close to the optimal performance in a homogeneous environment.

**Figure 7:**
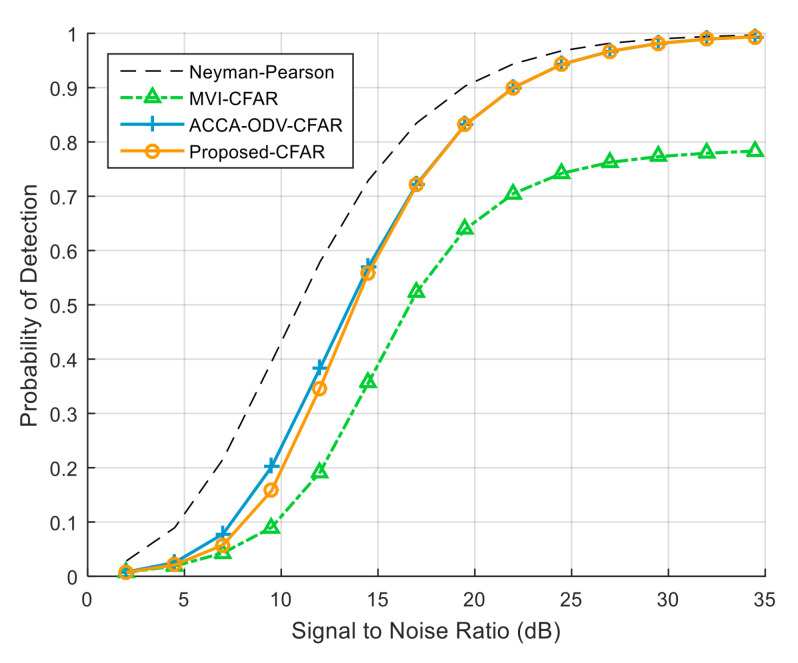
Detection probability on both sides of the reference window, where the numbers of interfering targets on both side of the reference window differ (*N* = 36, $P_{fa}$ = $10^{-4}$).

**Figure 8:**
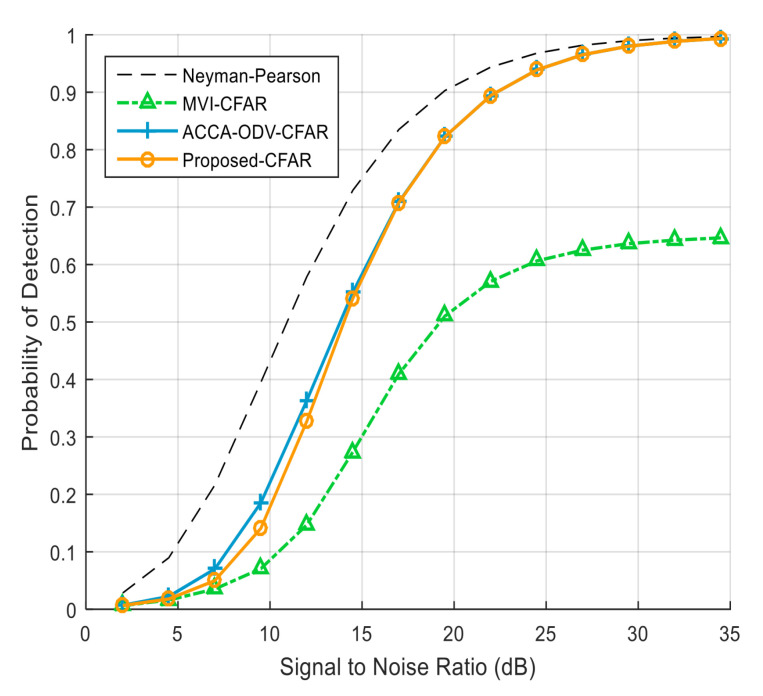
Detection probability on both sides of reference window and the number of interfering targets on both sides of reference window are the same (*N* = 36, $P_{fa}$ = $10^{-4}$).

Figure 7 exhibits the detection probability when the numbers of interfering targets in the leading and lagging windows are different; there are three and four interfering targets in the leading and lagging windows, respectively. Although the performance of the MVI CFAR algorithm degrades, the ACCA-ODV CFAR and proposed CFAR algorithms maintain a high detection probability. Figure 8 shows the detection probability when the numbers of interfering targets in the reference window on both sides of the test cell are the same, and there are four interfering targets in the leading and lagging windows. The MVI-CFAR algorithm produces a lower detection probability than when the numbers of interfering targets in the leading and lagging windows differ. In contrast, the ACCA-ODV CFAR algorithm and the proposed CFAR algorithm still maintain a high detection probability.

**Figure 9:**
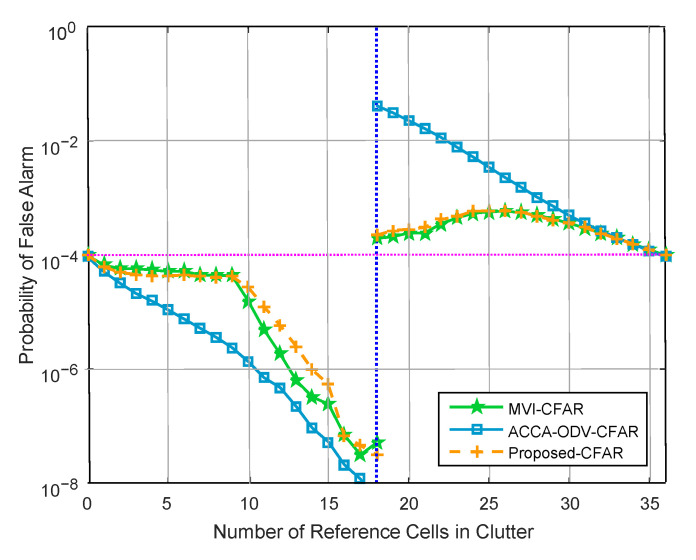
False alarm probability in clutter edge situation (*N* = 36, $P_{fa}$ = $10^{-4}$, and CNR = 10 dB).

Figure 9 shows the false alarm probability in a clutter edge environment. The false alarm probability of the ACCA-ODV CFAR algorithm rapidly increases when the test cell contains a clutter edge. This is because it censors until the dataset is determined to be homogeneous [20,21]. In contrast, the MVI and proposed CFAR algorithms show similar false alarm probabilities with the desired false alarm probability compared with that of the ACCA-ODV CFAR algorithm.

## 5. Hardware Architecture

**Figure 10:**
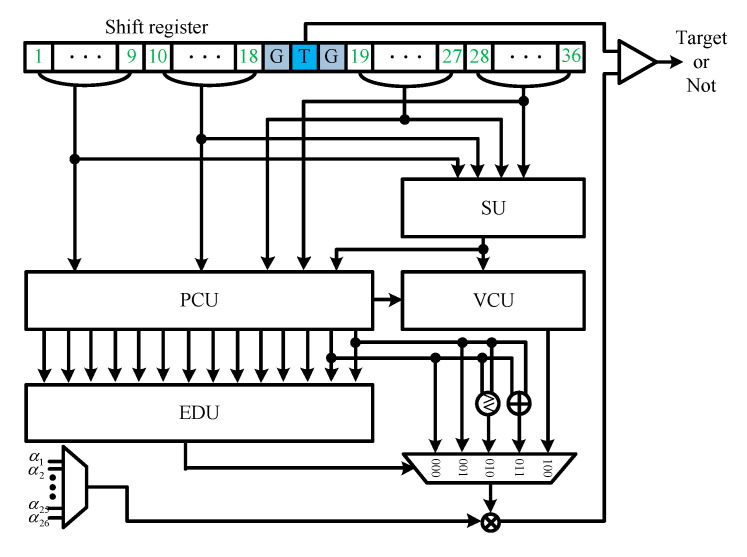
Hardware architecture of proposed CFAR algorithm.

The hardware structure of the proposed algorithm comprises a sorting unit (SU), parameter calculation unit (PCU), environmental decision unit (EDU), and $V_{k}$ comparator unit (VCU), as shown in Figure 10. When a new signal is input into the CFAR processor, the SU sorts the input data. Simultaneously, the input environment is determined by the PCU and EDU. The PCU calculates the necessary parameters when generating the environment identification index or performing the ACCA-ODV CFAR algorithm. The EDU generates an environment identification index through the parameters calculated by the PCU and determines the environment of the received signal. If two or more divided windows are deemed nonhomogeneous, a threshold is generated in the VCU using ACCA-ODV CFAR comparing $V_{k}$ with $S_{k}$. If fewer than two divided windows are determined to be nonhomogeneous, the GO or CA CFAR can be used. These generate thresholds using parameters calculated by PCU without a separate circuit.

### 5.1. Sorting Unit (SU)

**Figure 11:**
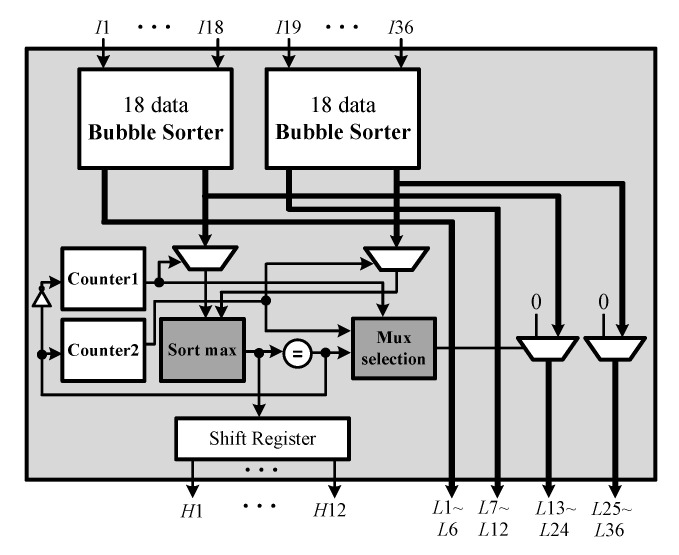
Hardware architecture of SU (*N* = 36; *p* = 24).

A general bubble sorter has a complex hardware architecture and a long execution time. In contrast, the ACCA-ODV CFAR algorithm does not sequentially sort all *N* data and only needs to sort the top *N*-*p* data. Therefore, we propose a sorter that satisfies the low-complexity and low-processing-time requirements by conducting bubble sorts with only a couple of data. The proposed sorter architecture is shown in Figure 11. After sorting 18 pieces of data, the value of the result is compared, and the top 12 pieces of data are determined, outputting them as H1,H2,…,H12, and L1,L2,…,L35, and L36. Because the top 12 data of each sorting result may be the top 12 data of the whole, the top 12 data are extracted by comparing each top 12 data. However, the lower six of each sorting result can never be the top 12 data, so they are output as L1,L2,…,L11, and L12. In addition, the output is 0 if the data are in the top 12 and is output as L13,L14,…,L35, and L36 if not in the top 12.

**Figure 12:**
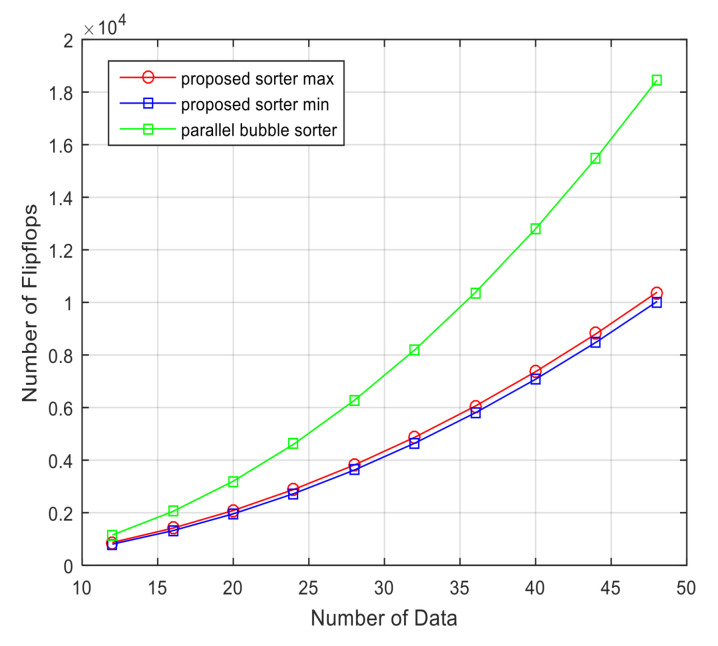
Comparison of the number of registers used according to the number of data of the received signal between the proposed sorter and general bubble sorter (input data length = 16).

**Figure 13:**
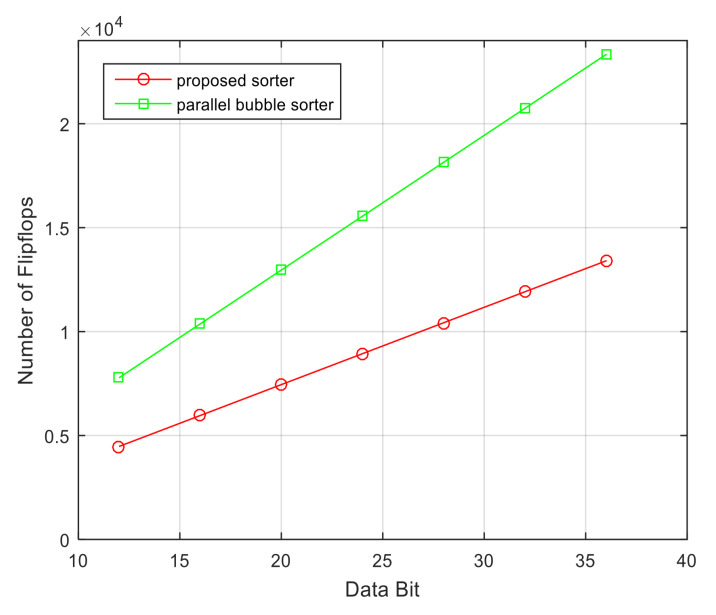
Comparison of the number of registers used according to the number of bits of the received signal between the proposed sorter and general bubble sorter (*N* = 36, *p* = 24).

Figure 12 shows the number of register usages of the parallel bubble sorter and the proposed sorter depending on the amount of received signal data. As the amount of data to be aligned increases, the deviation between the number of register usages of the proposed sorter and that of the bubble sorter increases. Figure 13 shows a graph comparing the number of register usages according to the number of bits in the received signal. As the number of bits of the received signal increases, the number of registers used by the parallel bubble sorter and the proposed sorter increases. However, the parallel bubble sorter uses approximately two times more than the proposed sorter.

### 5.2. Parameter-Calculation Unit (PCU)

**Figure 14:**
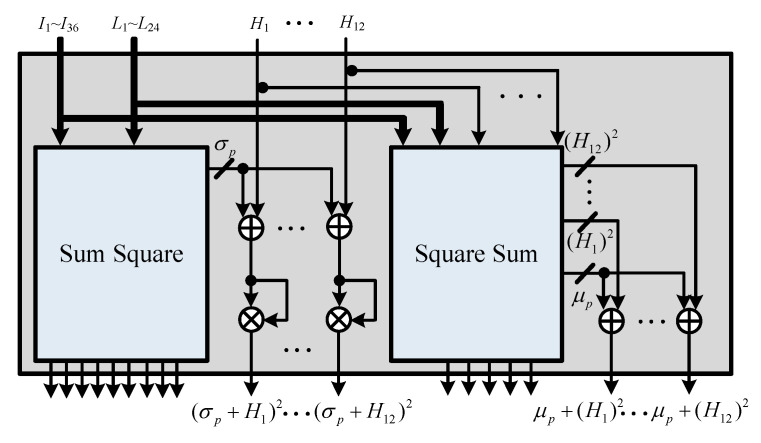
Hardware architecture of PCU.

**Figure 15:**
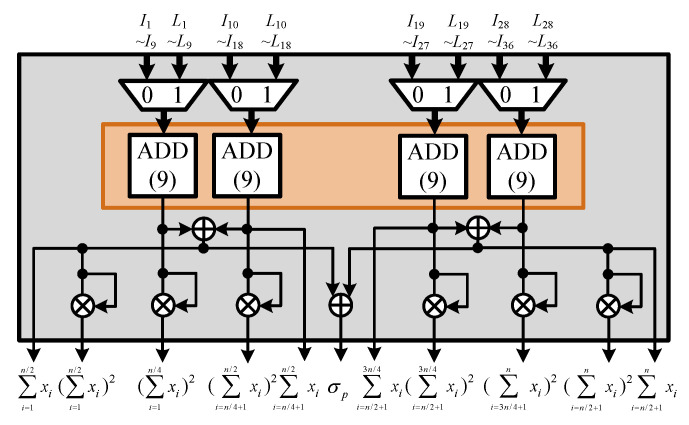
Hardware architecture of sum-square unit.

**Figure 16:**
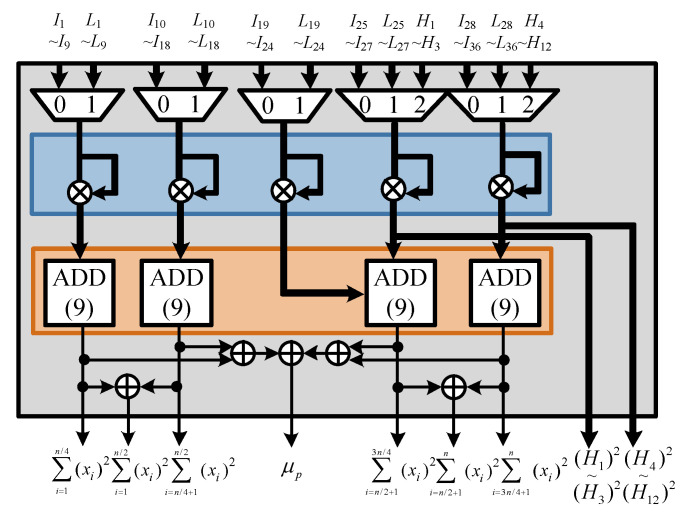
Hardware architecture of square-sum unit.

The proposed CFAR algorithm calculates the sum-square and square-sum operations when determining the received signal environment parameter. The ACCA-ODV CFAR algorithm executes the sum and square operations to determine the homogenous environment in the ordered data. The two operations are performed at different times because the environmental parameter of the received signal is generated immediately after the received signal is input. Homogeneity is determined after an alignment operation that takes a long time. Therefore, we designed an efficient hardware structure that shares the sum and square operations. The PCU is shown in Figure 14, where $\sigma_{p}$ and $\mu_{p}$ are calculated to perform the ACCA-ODV CFAR algorithm. In addition, square and sum operations are performed for the environmental judgment index operations. The structure of the PCU is divided into sum-square and square-sum units, and sum and square operations are performed. The structure of a PCU is divided mainly into sum-square and square-sum units, as shown in Figure 15 and Figure 16, respectively.

### 5.3. Environmental-Decision Unit (EDU)

**Figure 17:**
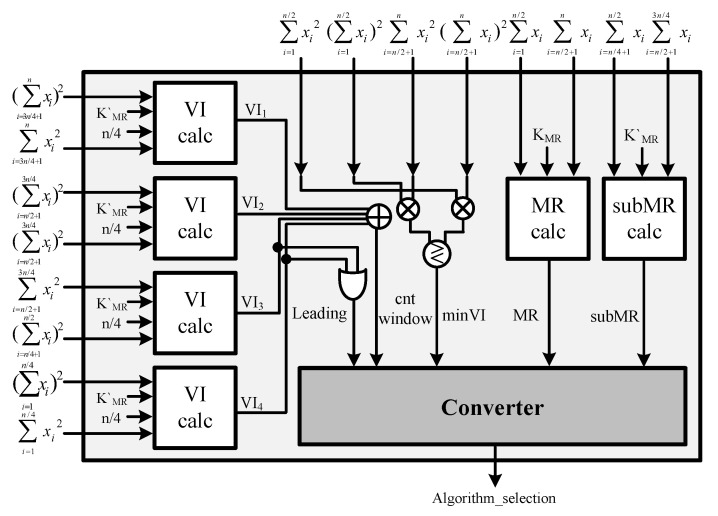
Hardware architecture of EDU.

**Figure 18:**
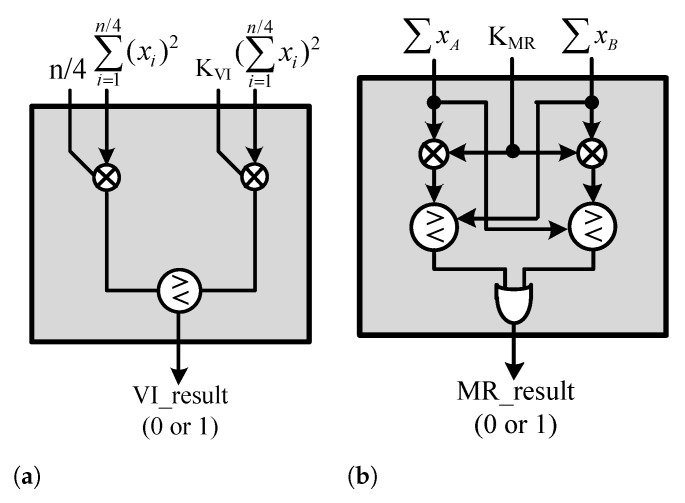
Hardware architecture: (**a**) VI-calculation Unit. (**b**) MR-calculation Unit.

The EDU calculates the indicators for determining the environment of the received signal and the number of windows determined to be nonhomogeneous; the structure is shown in Figure 17. To this end, the VI and MR calculation units are used. The VI calculation unit is shown in Figure 18a, and the MR calculation unit is shown in Figure 18b. The division is used as in Equation (17) when performing the VI operation. The equation is modified to reduce the hardware complexity as in Equation (18).

(17) $$MR=\frac{\sum_{i=1}^{\frac{N}{2}}x_{i}}{\sum_{i=1}^{\frac{N}{2}}x_{i}}>K_{MR}\;or\;\frac{\sum_{i=1}^{\frac{N}{2}}x_{i}}{\sum_{i=1}^{\frac{N}{2}}x_{i}}<K_{MR}^{-1}$$

(18) $$MR=\sum_{i=1}^{\frac{N}{2}}x_{i}>K_{MR}\times \sum_{i=1}^{\frac{N}{2}}x_{i}\;or\;\sum_{i=1}^{\frac{N}{2}}x_{i}<K_{MR}^{-1}\times \sum_{i=1}^{\frac{N}{2}}x_{i}$$

To avoid division even when performing MR operations, Equation (19) is transformed into Equation (20).

(19) $$VI=N\times \frac{\sum_{i=1}^{\frac{N}{2}}{\left(x_{i}\right)}^{2}}{{\left(\sum_{i=1}^{\frac{N}{2}}x_{i}\right)}^{2}}>K_{VI}$$

(20) $$VI=N\times \sum_{i=1}^{\frac{N}{2}}{\left(x_{i}\right)}^{2}>K_{VI}\times (\sum_{i=1}^{\frac{N}{2}}x_{i}{)}^{2}$$

### 5.4. $V_{k}$ Comparator Unit (VCU)

**Figure 19:**
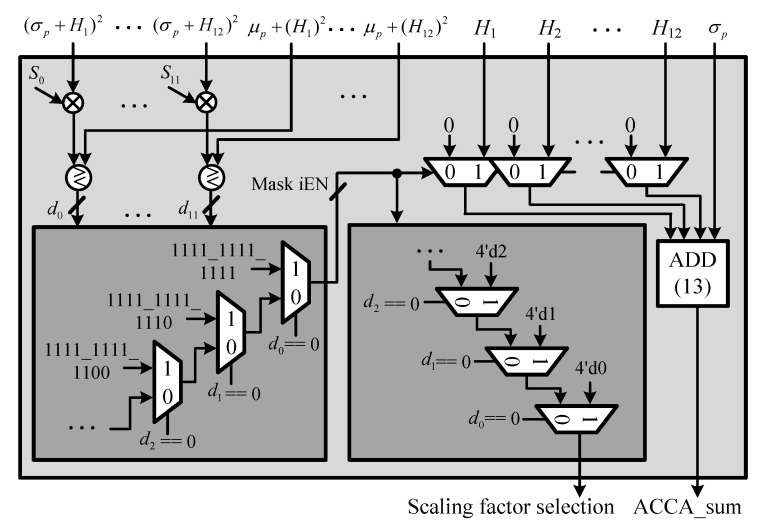
Hardware architecture of VCU.

The VCU computes $V_{k}$ and compares $V_{k}$ and $S_{k}$ when the ACCA-ODV CFAR algorithm operates, as shown in Equation (21). To reduce the hardware complexity, comparative Equation (21) is modified as shown in (22) to replace the division operation of $V_{k}$ with the multiplication operation, and the structure of the VCU is shown in Figure 19.

(21) $$V_{k}=\frac{\mu_{p}+{\left(X\left(N-k\right)\right)}^{2}}{{\left(\sigma_{p}+X\left(N-k\right)\right)}^{2}}>S_{k}$$

(22) $$V_{k}=\mu_{p}+{\left(X\left(N-k\right)\right)}^{2}>S_{k}\times {\left(\sigma_{p}+X\left(N-k\right)\right)}^{2}$$

To reduce the execution time, the comparison operation for each of $V_{0}$–$V_{\left(N-p\right)}$ and $S_{0}$–$S_{\left(N-p\right)}$ is performed in parallel: if true, 1 is output; if false, the result is 0. Thereafter, the dataset is censored by generating an enable signal of a multiplexer, as shown in Table 2, through $d_{0}$ to $d_{0}$–$d_{\left(N-p\right)}$. Finally, the censored dataset is combined and output.

### 5.5. Implementation and Results

**Figure 20:**
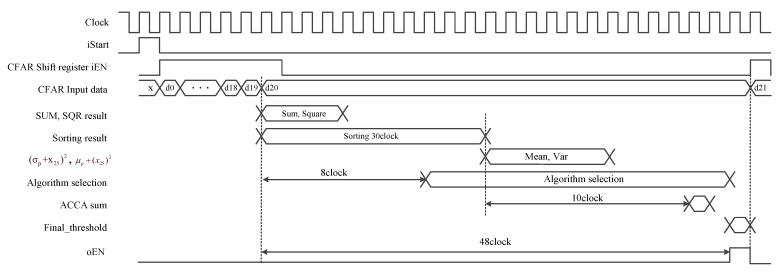
Timing diagram of the proposed CFAR processor.

We used the proposed CFAR processor configured a MATLAB-based simulator (The MathWorks Inc., Natick, MA, USA) to conduct a performance evaluation, and thenwe designed a fixed point for hardware implementation. Subsequently, it was designed at the register transfer level (RTL) using Verilog hardware description language (HDL). It was also implemented based on an Altera Stratix II EP2S60ES FPGA device and verified with test vectors from the MATLAB-based simulator for clutter edge and multitarget environments. Table 3 shows the synthesis results when the number of reference cells of the proposed CFAR processor was 16, and 8260 LUTs and 3823 registers were used. A timing diagram for the proposed processor is shown in Figure 20. It was confirmed that the maximum clock speed was 118.39 MHz and the processing time was 0.6 μs.

Table 4 shows a comparison of the results between the proposed method and other CFAR processors in [22,23,24,25,26,27,28,29,30,31]. For a fair comparison, we compared the speed performance in terms of the normalized operation time, $T_{norm}$, which was calculated based on FPGA process technology [32].

(23) $$T_{norm}=\frac{20}{Process}\times Operation\;time$$

An ACOSD CFAR-based algorithm was implemented in [22,23,24,25] for the case where the background distribution is log-normal. The CFAR processors in [22,25] have low complexity and fast operation time but cannot be applied to various environments, and a proposed method [23] has a problem with slow operation time. The CFAR processor presented in [24] has a fast operation time but has high hardware complexity in supporting various algorithms. One CFAR processor [26] can be applied to various environments with low hardware complexity but cannot be applied to real-time applications due to its long operation time. Another CFAR processor [27,28] can be applied to homogeneous and multitarget environments through CA, OS, and TM CFAR algorithms but experiences performance degradation in clutter edge environments. The CFAR processor in [29] has the advantages of low hardware complexity and fast operation time but requires prior information to generate thresholds. A CFAR processor that can be applied to Rayleigh distribution and no-Rayleigh distribution by applying mean level CFAR and log-t CFAR was presented [30]. However, it has disadvantages in terms of high hardware complexity and long operation time. The ACCA-ODV CFAR processor for multitarget environments was proposed [31], but its hardware complexity is very high. Moreover, because it supports only the ACCA-ODV algorithm, it is difficult to apply to various environments. Compared with previous implementation results, the proposed CFAR processor can provide superior performance for both homogeneous and nonhomogeneous environments using MVI and ACCA-ODV CFARs with the proposed decision criteria. In addition, even though the proposed CFAR processor can support both MVI and ACCA-ODV algorithms, the hardware complexity is very low and the operation speed is very high owing to the efficient hardware architecture presented in Section 3.

## 6. Conclusions

We developed an algorithm to determine the environment for performing appropriate CFAR. We also designed and verified the optimal hardware structure. The proposed algorithm divides the reference window into multiple numbers and performs the MVI and ACCA-ODV CFAR algorithms by determining whether the environment is non-homogeneous. The performance evaluation results showed high detection probability, such as the ACCA-OCV CFAR algorithm in multitarget environments, and low false alarm probability, such as the MVI CFAR algorithm in a clutter edge environments.

We reduced the hardware complexity with the time-sharing sum and square operations and by replacing division operations with multiplication operations when calculating decision parameters. We also proposed a low-complexity and high-speed sorter architecture that performs sorting for the partial data in leading and lagging windows. As a result, the implementation uses 8260 LUTs and 3823 registers and takes 0.6 μs to perform the operation. Compared with the previously proposed FPGA implementation results, we confirmed that the complexity and operation speed of the proposed CFAR processor are very suitable for real-time implementation. Therefore, it is expected to be suitable for systems that need to detect multiple targets and respond to clutter, such as radar systems for drone detection and radar systems for autonomous vehicles.

## Figures and Tables

**Table 1:** Simulation parameters.

Reference Window Size	Guard Cell Size	$P_{fa}$	$P_{fc}$	CNR	Number of Partition Windows	Size of Partition Window	Number of Trials	Noise Power
36	1	$10^{-4}$	$10^{-2}$	10 dB	4	9	$10^{6}$	1 dB

**Table 2:** Mask enable of VCU (*N* = 36, *p* = 24, ×: don’t care).

$d_{11}$	$d_{10}$	⋯	$d_{1}$	$d_{0}$	Mask Enable
×	×	⋯	×	0	0000_0000_0000
×	×	⋯	0	1	1000_0000_0000
		⋯			⋯
×	1	⋯	1	1	1111_1111_1110
1	1	⋯	1	1	1111_1111_1111

**Table 3:** Logic synthesis results of proposed CFAR targeting Stratix II (*N* = 16).

Unit	LUT	Registers
SU	3189	1846
PCU	3531	1351
EDU	579	105
VCU	371	194
Top Block	8260	3823
Fmax	118.39 MHz	

**Table 4:** Comparison of results between the proposed CFAR processor and the previous implementations.

Ref.	Platform	Algorithm	Resource		Fmax (MHz)	Operation Time	Process (nm)	Tnorm
			LUT	Register (Slices)				
[22]	Altera Stratix IV	ACOSD ^1^	4273	3074	250	0.45 μs	40	0.23 μs
[23]	Altera Stratix II	ACOSD ^1^	N/A	N/A	100	110 ms	90	24.44 ms
[24]	Altera Stratix IV	ACOSD ^1^, CA, OS	11,094	11,192	200	0.27 μs	40	0.16 μs
[25]	Xilinx Zynq 7000	ACOSD ^1^	10,441	12,688	148	0.24 μs	28	0.17 μs
[26]	Xilinx Virtex-IV	CA, GO, SO, OSCA ^2^, OSGO ^3^, OSSO ^4^	690	1364	N/A	84 ms	90	18.67 ms
[27]	Xilinx Virtex-IV	CA, OS	11,197	6027	59	N/A	90	N/A
[28]	Xilinx Virtex-6	CA, OS, TM	9272	5004	N/A	503 ms	40	251.5 ms
[29]	Xilinx KCU105	OS, OSCA ^2^, OSGO ^3^, OSSO ^4^	5697	2923	100	0.26 μs	20	0.26 μs
[30]	Xilinx Kintex-7	Mean Level, Log-t	99,650	27,082	100	83 μs	28	59.29 μs
[31]	Altera Stratix II	ACCA-ODV	18,861	5943	109.37	0.21 μs	90	0.05 μs
Proposed	Altera Stratix II	MVI, ACCA-ODV	8260	3823	118.39	0.6 μs	90	0.13 μs

^1^ Automatic censored ordered statistics detector, ^2^ order statistics cell averaging, ^3^ order statistics greatest of, and ^4^ order statistics smallest of.

## Data Availability

Not applicable.
